# Postmortem Analysis of Ethyl Alcohol Concentration in Blood, Urine, Muscle and Bile

**DOI:** 10.15388/Amed.2024.31.2.6

**Published:** 2024-12-04

**Authors:** Agnė Okulevičiūtė, Sigitas Chmieliauskas, Gabija Laubner-Sakalauskienė, Robertas Badaras, Sigitas Laima, Diana Vasiljevaitė, Jurgita Stasiūnienė

**Affiliations:** 1 Faculty of Medicine of Vilnius University, Vilnius, Lithuania; 2 Department of Pathology, Forensic Medicine, Institute of Biomedical Sciences of the Faculty of Medicine of Vilnius University, Vilnius, Lithuania.; 3 Department of Anaesthesiology and Intensive Care, Institute of Clinical Medicine of the Faculty of Medicine of Vilnius University, Vilnius, Lithuania.

**Keywords:** Ethyl alcohol, Forensic medicine, Postmortem alcohol production, Autopsy, Raktažodžiai: Etilo alkoholis, teismo medicina, toksikologija, autopsija

## Abstract

**Introduction:**

The determination of ethyl alcohol concentration in body fluids is an important investigation in forensic practice. To avoid postmortem changes in blood alcohol concentration, the test substance must be transported in special media enriched with sodium fluoride or potassium oxalate. When interpreting changes in concentrations in the body, it is important to assess not only the blood but also other body fluids or tissues.

**Materials and methods:**

A retrospective study was conducted from 2016 to 2023, evaluating data from nonconsecutive 378 autopsies from the State Forensic Medical Service of Lithuania. The study analyzed ethyl alcohol concentrations in blood, urine, muscle and bile. Toxicological data were processed using R commander statistical software. The study aimed to assess the changes, patterns, and correlations of ethyl alcohol concentrations in different body fluids after death.

**Results:**

When the ethyl alcohol concentrations of the different body fluids from the autopsies were evaluated, the urine ethyl alcohol concentration was in 86% cases higher than the blood ethyl alcohol concentration, with a mean difference of 0.51‰. There is a strong correlation between blood and urine ethyl alcohol concentrations, r = 0.93, p < 0.05. The ethyl alcohol concentration in muscle was 75% higher than in blood with a mean difference of 0.06‰. Ethyl alcohol concentration in bile was 90% higher than in blood with a mean difference of 0.14‰. The difference between ethyl alcohol concentrations in bile and muscle was not significant, with a mean difference of 0.07‰.

**Conclusions:**

In addition to blood and urine, muscle and bile samples may be taken at autopsy to detect ethyl alcohol. The results of the study show that there may be differences in the levels of ethyl alcohol in different body fluids after death. These data are therefore important for the assessment of ethyl alcohol concentrations in both clinical and forensic practice.

## Abstract (it)

**Įvadas:**

Etilo alkoholio koncentracijos nustatymas organizmo terpėse yra svarbus tyrimas teismo medicinos praktikoje. Siekiant išvengti pomirtinių alkoholio koncentracijos pokyčių kraujyje, tiriamoji medžiaga turi būti gabenama specialiose natrio fluoridu ar kalio oksalatu papildytose terpėse. Interpretuojant etilo alkoholio koncentracijos pokyčius organizme svarbu įvertinti ne tik kraują, bet ir kitus kūno audinių skysčius ar audinius.

**Medžiagos ir metodai:**

2016–2023 metais atliktas retrospektyvinis tyrimas, kurio metu buvo vertinami Valstybinės teismo medicinos tarnybos 378 autopsijų duomenys. Tyrimo metu buvo analizuota etilo alkoholio koncentracijos skirtinguose kūno skysčiuose ir audiniuose. Toksikologinių tyrimų duomenys buvo apdorojami statistine *R commander* programa. Tyrimu siekta įvertinti etilo alkoholio koncentracijos pokyčius, dėsningumus ir sąsajas skirtingose organizmo terpėse po mirties.

**Rezultatai:**

įvertinus autopsijų metu nustatytas etilo alkoholio koncentracijas skirtinguose kūno skysčiuose nustatyta, kad etilo alkoholio koncentracija šlapime 86 % buvo didesnė nei kraujyje – skirtumo vidurkis 0,51 ‰. Rasta stipri koreliacija tarp etilo alkoholio koncentracijos kraujyje ir šlapime r = 0,93, p < 0,05. Etilo alkoholio koncentracija raumenyje 75 % buvo didesnė nei kraujyje – skirtumo vidurkis 0,06 ‰. Etilo alkoholio koncentracija tulžyje 90 % buvo didesnė nei kraujyje – jų skirtumo vidurkis 0,14 ‰. Vertinant etilo alkoholio koncentracijų skirtumus tulžyje bei raumenyje, jų koncentracijos skyrėsi nereikšmingai – skirtumo vidurkis 0,065 ‰.

**Išvados:**

Autopsijos metu mėginiai etilo alkoholiui nustatyti gali būti imami ne tik iš kraujo ir šlapimo, tačiau gali būti vertinamas raumuo bei tulžis. Tyrimo rezultatai rodo, jog etilo alkoholio koncent-racijas skirtingose organizmo terpėse po mirties gali skirtis. Todėl šie duomenys svarbūs vertinant etilo alkoholio koncentraciją tiek klinikinėje, tiek ir teismo medicinos praktikoje.

## Introduction

Ethyl alcohol (EA) is one of the most important factors contributing to traffic accidents and disasters at work and home. It is also one of the most frequently analyzed substances in forensic medicine [1-3].

Blood is one of the most used human body fluids in which EA concentrations are analyzed, and to ensure accurate results, blood must be transported in a potassium oxalate (acting as an anticoagulant) and/or sodium fluoride (acting as a preservative) enriched medium to inhibit postmortem alcohol production [1,2,4]. Headspace gas chromatography is the method of choice worldwide for the qualitative and quantitative determination of ethanol in body fluids [1]. To ensure reliable results, it is recommended that blood is drawn from the femoral vein, as this is the least sensitive site for postmortem changes but other body tissues such as urine or vitreous fluid should also be collected to assess EA concentrations if possible [1]. Other body substances (urine, vitreous humor, bile, muscle) are important in the investigation of blood EA concentrations because they facilitate the identification of the presence of EA in the blood (premortem consumption of EA vs. postmortem increase of EA due to putrefactive processes), and EA, due to its low molecular weight and hydrophilicity, is distributed in water-rich tissues [2-5], therefore vitreous and urine with >95% water will have a higher concentration of EA than blood with 80–85% water (in the case of premortem alcohol consumption) [1,4,7].

## Materials and methods

### Study design and data source

In this retrospective study, we analyzed ethyl alcohol (EA) concentrations in various body fluids and tissues to determine patterns and correlations. From 2016 to 2023, 378 nonconsecutive autopsy cases where ethyl alcohol was detected in various body fluids and tissues were selected from the data of 1504 impersonalized autopsies. Cases where ethyl alcohol was detected only in blood or urine were excluded. All victims received full autopsies. Both blood and urine and other body substances (muscle and/or bile if possible) were collected and analyzed. Of the 378 cases, 14% (n=53) showed signs of decay.

### Identification of cases

The State Forensic Medicine Service (Lithuania) provided the autopsy data for 378 cases. Toxicological tests for alcohol and drugs were done for all cases. In every case, information was provided by the law enforcement agencies, including the possible crime location, time of death, and presumed death mechanism.

### Limitations

The population of Lithuania served as the study’s subject; hence, the results cannot accurately reflect the condition of the entire global population. In our study, we only analyzed autopsy data. The exclusion criterion was age less than 18, and people who had been treated in hospital before death.

### Statistical analysis

The collected data was processed using R software. The Shapiro–Wilk test was used to determine whether the data was normally distributed. The Student’s t-test was used to assess the statistical significance of differences in continuous variables between the study groups. Spearman’s correlation coefficients were assessed. A weak correlation was defined as R-values ≤ 0.39; a moderate correlation with R-values from 0.40 to 0.69; and a strong correlation with R-values≥ 0.70. Differences with p-values less than 0.05 were considered significant.

### Toxicological analysis

After the forensic dissection, blood and urine samples were collected for alcohol and drug tests. Headspace gas chromatography was used to detect the presence of alcohol, while liquid chromatography-time-of-flight mass spectrometry (LC/MS-TOF) and chromatography-tandem mass spectrometry (LC-MS/MS) were used for quantitative drug detection.

## Results

The mean age of the whole sample was 53.91 ± 14.14 years (median 55 years). Mean age was 52.34 ± 13.57 years for males and 59.53 ± 15.02 years for females, with statistically significant differences p < 0.05.

The mean ethyl alcohol concentration in the blood of the deceased was 1.86 ± 1.27‰ (median 1.76‰). The lowest concentration in the sample was 0.15‰ and the highest was 6.1‰. The median ethanol concentration in the blood of males and females was statistically insignificant (p = 0.41).

The mean ethyl alcohol concentration in the urine of the deceased was 2.49 ± 1.40‰ (median 2.54‰). The lowest concentration was 0.16‰, the highest 6.66‰. The median ethyl alcohol concentration in the urine of males and females was statistically insignificant, p = 0.67.

**Table 1:** When the ethyl alcohol concentration of the deceased was determined in both blood and urine together

	Total	Male	Female
Number of case (N)	378	319	59
Mean BAC and SD	1.86 ± 1.27‰	1.88 ± 1.2 6‰	1.79 ± 1.32‰
Mean UAC and SD	2.49 ± 1.40‰	2.48 ± 1.39‰	2.55 ± 1.44‰

Abbreviations: BAC – blood alcohol concentration; UAC – urine alcohol concentration; SD – standard deviation

**Fig. 1:**
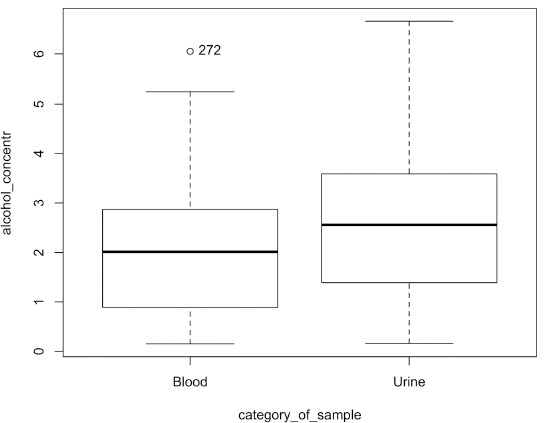
Ethyl alcohol concentration in blood and urine

The mean difference between blood and urine ethanol concentrations was 0.51 ‰ (median difference 0.46 ‰). During the comparison of blood and urine ethyl alcohol concentrations, we found that the urine ethyl alcohol concentration was in 86% cases higher than the blood one, while only in 14% cases the blood alcohol concentration was higher than the urine one. The difference between blood and urine ethyl alcohol concentrations was statistically significant at p < 0.05 (Fig. 1).

The Spearman’s rank correlation between blood and urine ethyl alcohol levels (r = 0.93, p < 0.05) shows that there is a very strong statistically significant correlation between the two media.

The correlation of the age of the deceased with blood ethyl alcohol concentration yielded a negative result of r = –0.14, p < 0.05, indicating that there is a weak negative statistically significant correlation between age and blood ethyl alcohol concentrations. This means that as age increases, ethyl alcohol concentration decreases.

When ethyl alcohol concentration was determined in the lumbar striated muscle of the deceased, the mean concentration was 0.86 ± 0.85‰ (median 0.53‰). The lowest ethyl alcohol concentration found in the muscle was 0.15‰ and the highest was 4.73‰. The difference of median ethyl alcohol concentrations in the muscle of males and females was statistically insignificant, p = 0.41.

**Table 2:** When the ethyl alcohol concentration of the deceased was determined in both blood and muscle together

	Total	Male	Female
Number of case (N)	177	150	27
Mean BAC and SD	0.69 ± 0.66‰	0.73 ± 0.72‰	0.57 ± 0.38‰
Mean MAC and SD	0.86 ± 0.85‰	0.95 ± 0.94‰	0.73 ± 0.62‰

Abbreviations: BAC – blood alcohol concentration; MAC – muscle alcohol concentration; SD – standard deviation

**Fig. 2:**
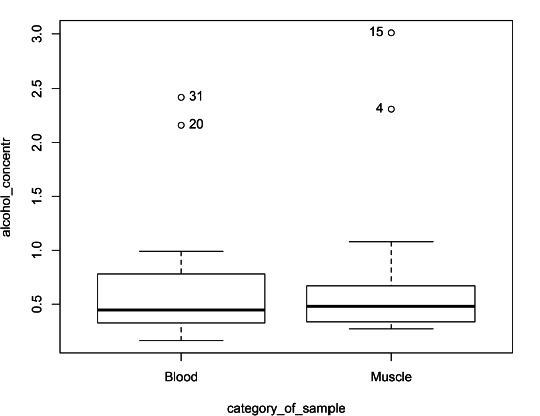
Ethyl alcohol concentration in blood and muscle

It is important to check how the muscle ethyl alcohol concentration differs from the blood concentration. When the ethyl alcohol concentration of the deceased was determined in both blood and muscle together, the mean difference of ethyl alcohol concentration was 0.06‰ (median difference 0.07‰). When comparing blood and muscle ethyl alcohol concentrations, 75% of the cases had a higher ethyl alcohol concentration in the muscle but the difference was statistically insignificant at p = 0.78 (Fig. 2).

The Spearman’s rank correlation (r = 0.47, p = 0.06) shows that there is a statistically unreliable but moderate correlation between the two media.

The mean concentration of ethyl alcohol in bile is 1.06 ± 1.02‰ (median 0.71‰). The median ethyl alcohol concentrations in the bile of males and females differed statistically insignificantly, at p = 0.29.

**Fig. 3:**
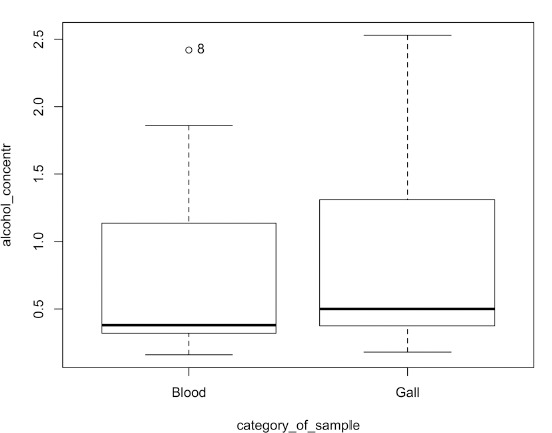
Ethanol concentration in blood and bile

When ethyl alcohol concentrations were determined in the blood and bile of the deceased, the mean difference of ethyl alcohol concentration was 0.14‰ (median difference 0.07‰). When comparing ethyl alcohol concentrations in blood and bile, 90% of cases had higher ethyl alcohol concentrations in bile, although the difference in concentrations was statistically insignificant at p = 0.41 (Fig. 3).

When ethyl alcohol concentrations were determined in the muscle and bile of the deceased, the mean difference of ethyl alcohol concentrations in these two matrices was 0.065‰ (median difference 0.03‰).

**Fig. 4:**
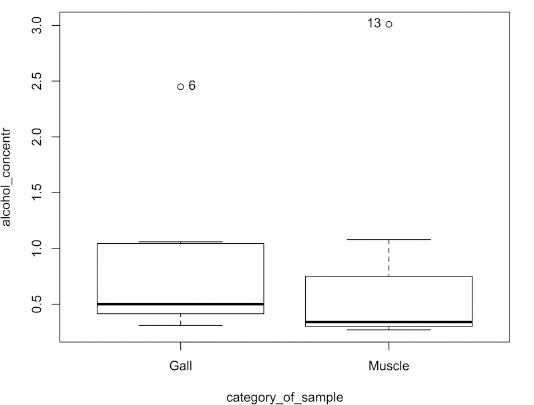
Ethyl alcohol concentration in muscle and bile

When comparing the ethyl alcohol concentrations in bile and muscle, 57% of the cases had higher ethyl alcohol concentration in bile, although the difference was statistically insignificant at p = 0.38 (Fig. 4).

The difference between the median ethyl alcohol concentrations in muscle and bile was statistically insignificant, p = 0.44.

The group with higher urine than blood ethyl alcohol concentrations had higher muscle and bile ethyl alcohol concentrations compared to the group with higher blood than urine ethyl alcohol concentrations, although the difference was not statistically significant, p > 0.05.

The ethyl alcohol concentrations of the deceased with and without signs of decay differed statistically insignificantly by p > 0.05 in all tested media, i.e. in blood p = 0.09, in urine p = 0.21, in muscle p = 0.24 and in bile p = 0.42.

**Table 3:** When the ethyl alcohol concentration of the deceased was determined in both blood and bile together

	Total	Male	Female
Number of case (N)	77	50	27
Mean BAC and SD	0.84 ± 0.74‰	0.98 ± 0.80‰	0.47 ± 0.45‰
Mean bile AC and SD	1.06 ± 1.02‰	1.22 ± 1.13‰	0.59 ± 0.31‰

Abbreviations: BAC – blood alcohol concentration; bile AC – bile alcohol concentration; SD – standard deviation

Moreover, when assessing the correlation between ethyl alcohol concentrations in blood and bile (Spearman’s rank correlation), the result was r = 0.85, p < 0.05, indicating that there is a statistically significant strong correlation between the ethyl alcohol concentrations in these two mediums.

The Spearman’s rank correlation of the ethyl alcohol concentrations in muscle and in the bile yielded a result of r = 0.78, p < 0.05, indicating that a statistically reliable strong correlation between the concentrations in the two media is observed.

The Spearman’s rank correlation of the ethyl alcohol concentrations in the urine and in the bile yielded a result of r = 0.31, p = 0.37, indicating a statistically unreliable moderate-weak correlation between the concentrations in these two media.

## Discussion

In practice, forensic pathologists often have to answer the question of whether ethyl alcohol was administered before or after death [5]. The estimation of EA in the subject’s body can be complicated by the fact that decaying processes may produce alcohol after death, as microorganisms can produce ethyl alcohol from glucose [4–6]. The postmortem estimation of EA concentration is highly dependent on the trauma (contamination), the time of decomposition of the body, the sampling technique, and the storage conditions of the deceased (if the corpse is kept in a refrigerator for the first 24 h, it is assumed that postmortem EA production does not occur) [1,4,5,7]. The diseases of the deceased are also relevant – diabetes mellitus can lead to higher blood glucose concentrations, which will increase the amount of substrate available for microorganisms after death [1,3,5].

In healthy people, glucose should not be found in the urine, therefore the postmortem finding of EA in the urine suggests that alcohol was consumed before death [4,8]. Difficult assessment of urinary EA may occur if the deceased had diabetes mellitus due to glycosuria [1,4].

The vitreous humor is also suitable for the assessment of EA concentrations because of the easy accessibility of the specimen, low likelihood of diffusion of EA from the intestine, low likelihood of contamination with micro-organisms, and very rapid equilibrium of the concentration between vitreous humor and blood [2,4,6,7,9]. A statistically reliable correlation between EA levels in the blood and vitreous fluid has also been demonstrated [6,9].

Bile may be an alternative test substance in forensic medicine because of its ease of collection, the large volume of the test substance that can be collected, and the accumulation of high concentrations of psychoactive substances and their metabolites, but bile, like the aforementioned liquids, is more suitable as a qualitative rather than a quantitative test for the assessment of EA concentrations [10].

If the body is in an advanced state of decomposition, muscle tissue may also be used to assess EA concentrations, as it is less likely to produce EA postmortem [1,5].

When analyzing blood levels of EA, it is also important to consider the metabolites of ethanol degradation. Ethyl glucuronide (EG) is a direct metabolite of ethanol degradation found in urine. This breakdown product is important because it can be detected several days after alcohol consumption and is also detectable at even low concentrations of EA, but it has limited stability in severely decayed bodies [1,6]. However, if elevated concentrations of EA in the blood and no degradation metabolites are found, it can be assumed that the alcohol has been produced postmortem [4,11]. Analysis of serotonin metabolites in urine and calculation of the 5-hydroxytryptophol/5-hydroxyindoleacetic acid (5-HTOL/5-HIAA) ratio is another well-established method to determine the source of ethanol in the blood after death. Acetaldehyde formed during ethanol metabolism competes with the intermediate aldehyde involved in serotonin metabolism, therefore when 5-HTOL/5-HIAA in the urine of a deceased person is >15, it is reliable evidence that the person had consumed alcoholic beverages prior to death [4,11]. Autopsy examination of the deceased’s tissues may reveal higher alcohols produced by fermentation by microorganisms (mainly 1-propanol), which would suggest that ethanol was produced after death [1,3].

According to studies, an endogenous (microorganism-mediated) increase in EA levels can be considered as >10 mg/dl when EA is present in the blood but not in vitreous humor or urine [5]. In all other cases, if the individual has been in the absorption phase of EA, the blood EA concentration will likely be higher than the vitreous humor concentration, which respectively will be higher than the urine concentration. According to more recent literature, difficulties arise when the EA blood concentration is below 40 mg/dl, in which case, in the absence of an alternative sample or evidence collected, it is reasonable to predict that the 40 mg/dl blood alcohol concentration is due to predeath alcohol consumption [5].

### Pharmacokinetics

Dosage, beverage type, drinking speed, and individual characteristics such as age and body weight can have an impact for blood-alcohol concentration (BAC). Once absorbed ethanol mainly acts as a central nervous system (CNS) depressant. Ethanol’s effects may vary from mild euphoria at lower BACs to severe mental impairment and risk of coma or death at higher levels, predominantly because of its interaction with GABA-A receptors and inhibition of glutamate receptors in the CNS [12,13].

Initial absorption (about 80%) of ethanol takes place in the small intestine, and the remainder occurs in the stomach. This absorption is regulated by the pyloric sphincter and results in first-pass metabolism in the liver, where approximately 20% of ethanol is metabolized before entering systemic circulation. Ethanol, as a small uncharged molecule, follows zero-order kinetics during elimination, therefore a constant amount is removed from the blood per unit time, and its rate of absorption and metabolism is influenced by factors such as stomach contents and drinking rate [13,14].

After absorption and undergoing first-pass metabolism, ethanol is distributed throughout the body, including the brain and skeletal muscle, with its volume of distribution closely related to total body water (TBW). Ethanol’s distribution varies by gender and age, typically 0.6 L/kg in women and 0.7 L/kg in men, reflecting differences in TBW. Ethanol concentrates more in biofluids with high water content, such as sweat, saliva, and urine, compared to blood, serum, or plasma, with distribution studies confirming its correlation with TBW [13,14].

Primary metabolism of ethanol happens in the liver, where alcohol dehydrogenase (ADH) converts ethanol to acetaldehyde, which is then further oxidized to acetic acid by aldehyde dehydrogenase (ALDH). During this process, energy is produced, which increases the NADH/NAD+ ratio, and can lead to metabolic disturbances such as lactic acidosis and gout [13].

Most ethanol is eliminated from the body through oxidative metabolism in the liver, with less than 10% excreted unchanged via the lungs, kidneys, and skin. After moderate drinking, 2–5% of the consumed ethanol can be detected in urine, exhaled air, and sweat, with higher concentrations expected at elevated blood alcohol levels. The overall blood ethanol elimination rate ranges from 0.10 to 0.25 g/L (h) [13,14].

Analyzing alcohol levels in blood and consecutive urine samples is effective for detecting recent drinking. While increased urine alcohol concentration (UAC) between two voids suggests recent consumption, a decreasing UAC indicates the postabsorptive phase. Urine is produced at about 1 mL per minute, with the UAC typically being 1.25 times higher than blood alcohol concentration (BAC) due to differences in water content. BAC can also fluctuate rapidly based on alcohol absorption and metabolism [15].

## Conclusion

Proper assessment of postmortem ethyl alcohol levels necessitates venous blood collection and transport in sodium fluoride-containing media, alongside the analysis of urine, especially in cases where the deceased did not suffer from diabetes mellitus. Additionally, the examination of other tissues can provide valuable insights into the timing of alcohol consumption. Forensic practice may involve the analysis of breakdown products of ethyl alcohol to ascertain antemortem alcohol consumption. Our study underscores significant differences in EA concentrations among different body fluids and tissues, with urine consistently displaying higher levels compared to blood. Furthermore, we observed a robust correlation between blood and urine EA concentrations, validating the utility of urine analysis in detecting premortem alcohol consumption.
